# Predation impact on threatened spur-thighed tortoises by golden eagles when main prey is scarce

**DOI:** 10.1038/s41598-022-22288-9

**Published:** 2022-10-25

**Authors:** José M. Gil-Sánchez, Roberto C. Rodríguez-Caro, Marcos Moleón, María C. Martínez-Pastor, Mario León-Ortega, Sergio Eguía, Eva Graciá, Francisco Botella, José A. Sánchez-Zapata, Julia Martínez-Fernández, M. A. Esteve-Selma, A. Giménez

**Affiliations:** 1 Department of Zoology, University of Granada, Avda. de Fuente Nueva, s/n, 18071 Granada, Spain; 2 Department of Applied Biology, Miguel Hernández University, Avda. de la Universidad, s/n, 03202 Elche, Spain; 3 Department of Zoology, Oxford University, 11a Mansfield Road, Oxford, OX1 3SZ UK; 4 Department of Ecology, University of Alicante, Carr. de San Vicente del Raspeig, s/n, 03690 San Vicente del Raspeig, Alicante, Spain; 5 Ulula Asociation: Nocturnal Bird Monitoring, C/Herreras y Moreras. 12, 30110 Churra, Murcia, Spain; 6 MENDIJOB, S.L., c/Rambla, 22, 30120 El Palmar, Murcia, Spain; 7 Centro de Investigación e Innovación Agroalimentaria y Agroambiental (CIAGRO-UMH), Miguel Hernández University, Carretera de Beniel Km 3.2, 03312 Orihuela, Spain; 8 Fundación Nueva Cultura del Agua, C/ Pedro Cerbuna, 12, 50009 Zaragoza, Spain; 9 Department of Ecology and Hydrology, University of Murcia, Campus de Espinardo, 30100 Murcia, Spain

**Keywords:** Ecology, Zoology

## Abstract

A reduction in adult survival in long-living species may compromise population growth rates. The spur-thighed tortoise (*Testudo graeca*) is a long-lived reptile that is threatened by habitat loss and fragmentation. Golden eagles (*Aquila chrysaetos*), whose breeding habitats overlap that of tortoises, may predate them by dropping them onto rocks and breaking their carapaces. In SE Spain, the number of golden eagles has increased in the last decades and the abundance of their main prey (i.e., rabbits *Oryctolagus cuniculus*) has decreased. Our aims were to 1) describe the role of tortoises in golden eagles’ diet, and 2) estimate the predation impact of golden eagles on tortoises in eagles’ territories and in the regional tortoise population. We collected regurgitated pellets and prey remains under eagle nests and roosts, and obtained information on tortoise abundance and population structure and rabbit abundance. We found that tortoises were an alternative prey to rabbits, so that eagles shifted to the former where the latter were scarce. The average predation rate on tortoises was very low at the two studied scales. However, eagles showed a marked selection for adult female tortoises, which led the tortoise sex ratio to be biased towards males in those eagle territories with higher tortoise predation. Whether this may compromise the spur-thighed tortoise long-term population viability locally deserves further attention.

## Introduction

Adult survival, i.e. the survival of breeding individuals, is a key vital rate in the demography of long-living species^1,2^. These species usually display a demographically buffered strategy^3^, with little variation in the vital rates that most affect population growth rates^4^. Thus, for long-living species, even weak fluctuations in chief demographic traits can affect their population dynamics, which may lead to population declines or even local extinctions^5^. This is highly relevant for biodiversity conservation, as many globally threatened species in all ecosystems are long-living^6,7^. Therefore, understanding the drivers of adult survival in long-living species is of paramount importance in the current scenario of environmental change.

Destabilising variations in adult survival rates can result from predation under exceptional conditions, such as the presence of invasive predators^8^ and following stochastic environmental changes^4^. Generalist predators can also enhance predation pressure on alternative prey when the population of their primary prey species is depleted and/or when the predator population increases, thus leading to a hyperpredation phenomenon^9–14^. In turn, hyperpredation can drive both local- and large-scale declines in prey^9,13,15^, which can be especially dramatic for small populations of endangered species.

**Figure 1:**
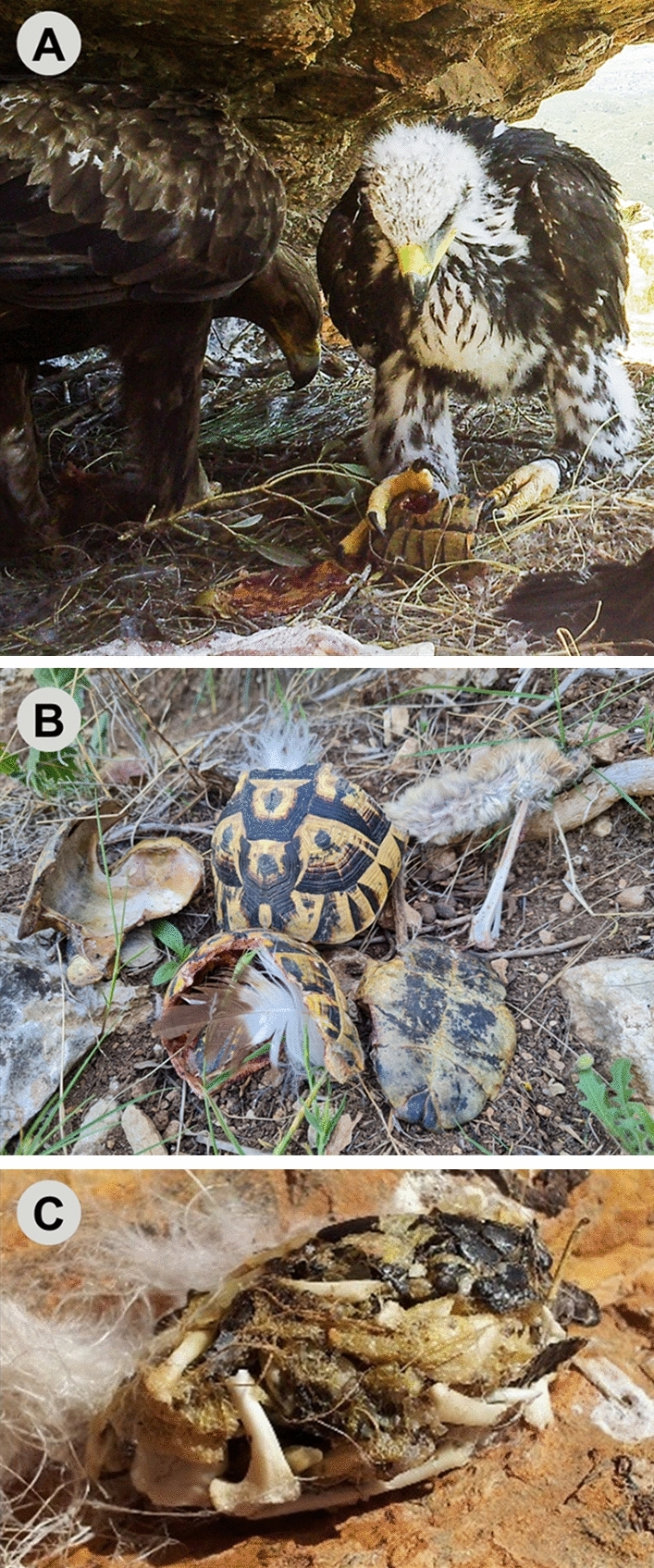
Golden eagles *Aquila chrysaetos* have developed an elaborate technique to handle non-juvenile, hard-carapaced tortoises, consisting of lifting the tortoise and dropping it onto rocks to break open the hard shell. (**A**) A golden eagle nest (adult and juvenile individuals) with spur-thighed tortoise *Testudo graeca* remains in SE Spain (photo: Mario León-Ortega). (**B**) Broken carapaces of spur-thighed tortoise below a golden eagle nest in SE Spain (photo: Andrés Giménez). (**C**) A golden eagle pellet, consisting entirely of spur-thighed tortoise remains, from an eagle roost in SE Spain (photo: Marcos Moleón).

Tortoise populations of the family Testudinidae, which includes typically long-living species, are extremely vulnerable to enhanced adult mortality^16,17^. Once tortoises have fully developed their hard shell, predation of adult individuals is rare. This makes tortoises one of the most long-lived animals, with losses to predators mainly limited to juveniles^18^. Strikingly, golden eagles (*Aquila chrysaetos*), the paradigm of generalist avian predators^19^, are able to successfully feed on adult terrestrial tortoises. They do so using an elaborate hunting technique consisting of lifting a tortoise and dropping it onto rocks to break open its hard shell^19^, a practice that has been described since ancient Greek times (450 BC)^20^ (Fig. 1). Golden eagles have been cited to prey on four tortoise species from the Mediterranean Basin to eastern Asia: *Testudo graeca*^21–24^; *T. hermanni*^22,25,26^; *T. marginata*^22^; and *T. horsfieldii*^27^. In some areas, tortoises can represent up to c. 90% of the golden eagle’s diet^28^. This indicates that predation by golden eagles on tortoises is common wherever they coexist in the Western Palearctic^29^. Although some studies have described the role of tortoises in eagles’ diet, the potential effects on tortoise populations have not been previously evaluated.

**Figure 2:**
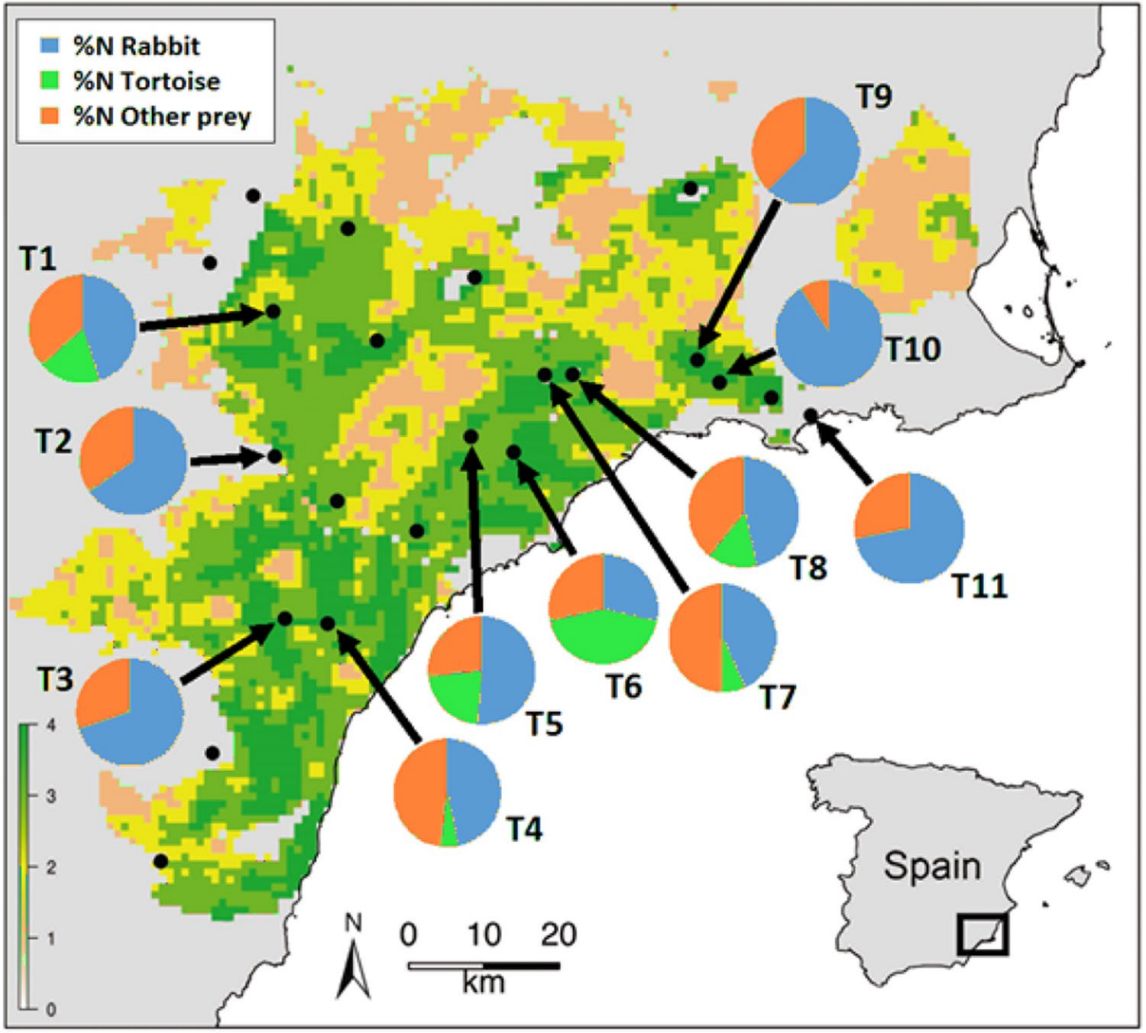
Study area. The abundance of spur-thighed tortoise is shown for each 1 × 1 km cell with tortoise presence, according to four density classes^70^: 1 = very low (< 0.07 ind./ha), 2 = low (0.07–0.42 ind./ha), 3 = medium (0.42–2.66 ind./ha), and 4 = high density (> 2.66 ind./ha). The map with the densities, with modification, is reprinted with permission from the author and the publisher^69^. Dots indicate golden eagle territories. Pie charts show the diet of the golden eagle territories with sufficient diet data (i.e. > 20 prey).

The spur-thighed tortoise (*T. graeca*) is a globally endangered species (Vulnerable)^30^ that is widely distributed across N Africa, S Europe and SW Asia^31^. Its largest population in W Europe is found in SE Spain (Fig. 2)^32,33^. In SE Spain, the area inhabited by this medium-sized terrestrial tortoise is mainly characterised by a mosaic of semiarid shrublands, non-irrigated crops (e.g. almond and olive groves) and abandoned crops^34^, where tortoise’s populations reach similar densities^35^. This species is characterised by slow population dynamics^36^, steady population growth rates^37,38^, delayed maturation (9–12 years)^17^, and low offspring production (< 2 hatchings per female and year)^39^. In SE Spain, it is heavily threatened by habitat loss and fragmentation^35,40^, also due to its relevance in the pet trade^41^. Spain is home to a large population of golden eagles that partially overlaps with the tortoise population range^42^. Here, golden eagle predation on spur-thighed tortoises is known to occur^21,23^, although this has not been studied in detail.

Golden eagles and spur-thighed tortoises have long coexisted in SE Spain, probably since the last glacial age some 20,000 years ago^33^. This suggests that the tortoise population is resilient to the effect of eagle predation. However, in recent decades, the population of the golden eagle’s staple prey in Iberian ecosystems, the European rabbit (*Oryctolagus cuniculus*), has suffered a severe decline caused by two emergent viral diseases: myxomatosis, in the mid-twentieth century, and rabbit haemorrhagic viral disease (RHD) since the 1990s^43^. After these rabbit disease outbreaks, golden eagles shifted their diet to secondary prey, such as other mammals, birds and reptiles^23,44^, as was the case for other Iberian predators^45–47^. In addition, despite diminished rabbit abundance, the golden eagle population has notably increased due to conservation actions during the last decades in Spain, including the distribution range of the spur-thighed tortoise^42^ (authors’ unpublished data). Thus, the increase in predator abundance (golden eagle), along with the pronounced decrease in the main prey (rabbit) population, could promote hyperpredation on secondary prey (spur-thighed tortoise)^13^.

Here, we assess the golden eagle-tortoise relationship, focussing on the native spur-thighed tortoise population in SE Spain. Due to its threatened status^30^, the need for detailed study of this novel predator–prey interaction scenario is urgent. Moreover, this interaction may pose a conservation dilemma, as both are protected species in Europe. The main goals of this study were to: 1) describe the role of the spur-thighed tortoise in the golden eagle’s diet, including its relative importance to prey selection, the size, stage and sex of tortoises selected by eagles, and functional response of eagles in consumption of tortoises with changing prey densities; and 2) estimate the predation impact of golden eagles on tortoises at two spatial scales (eagles’ territories and total tortoise population). We hypothesised that the impact of predation on tortoises would be influenced by the availability of the predator’s staple prey, namely the European rabbit. Ultimately, we aimed to unravel the limiting and regulating potential of a versatile predator on a prey population of a long-living species of conservation concern that is generally subject to low adult predation rates.

## Results

### Golden eagle diet and preferred prey

We found 496 pellets that contained 811 individuals from 20 prey species in 11 golden eagle territories (50% of territories within the study area; see Supp. Mat. Table S1 for details), namely those territories in which access to nesting and roosting sites–and, therefore, to pellets and prey remains–was possible. The sampled territories are a good representation of the habitat and altitudinal variation in the study area. The golden eagle’s diet was composed primarily of rabbits, which represented 54.2%N and 61.9%B on average. Other mammals (especially juvenile wild boars *Sus scrofa*), red ledged partridges (*Alectoris rufa*), other birds (mainly pigeons), tortoises (11.5%N, 7.4%B) and other reptiles (mainly snakes) each comprised less than 12%N and %B. Some inter-territorial differences were found in diet, but rabbits were the main prey in most cases (Fig. 2). Tortoises were consumed in six golden eagle territories (54.5% of the territories with at least 20 prey), with tortoises being the most frequently consumed prey in one territory (42.9%N; T6 in Fig. 2).

**Figure 3:**
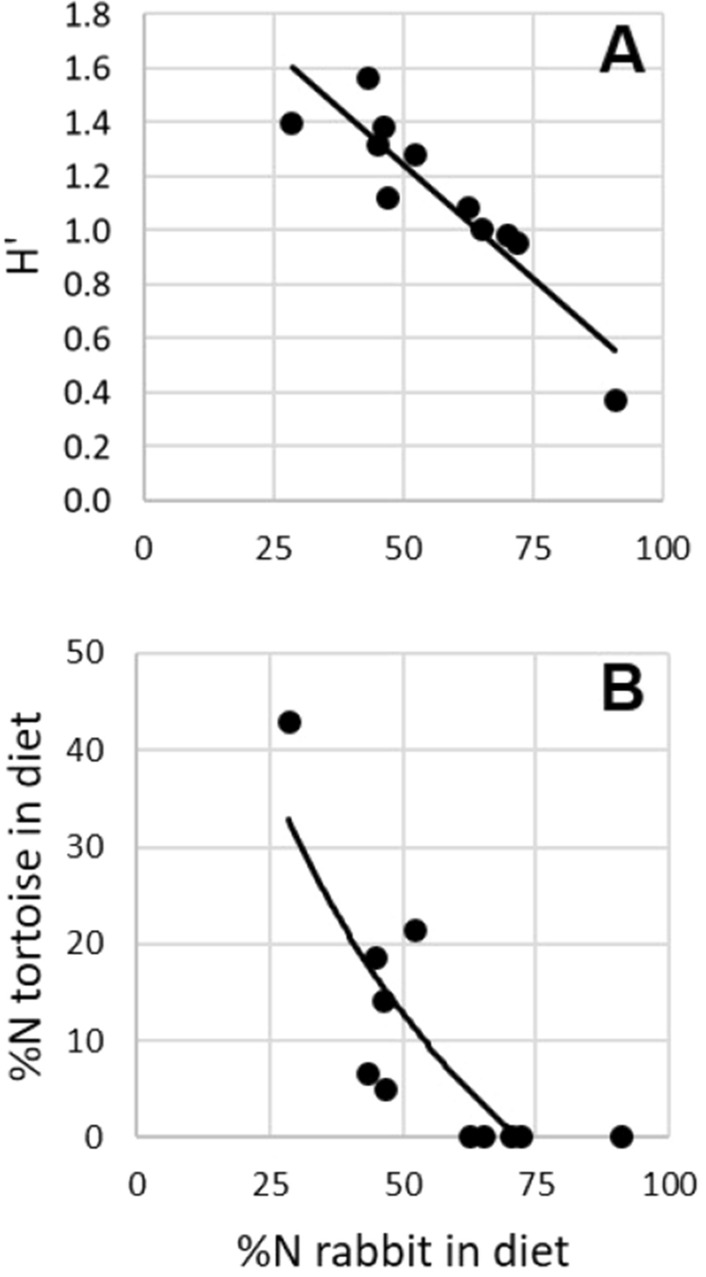
Relationship between the consumption of rabbits and (**A**) eagles’ trophic diversity (Shannon–Weaver index, H’) and (**B**) tortoise consumption in the 11 golden eagle territories of the study area with sufficient diet sample size. In both cases, the inverse relationship was significant (see text for more details).

The consumption (%N) of rabbit was negatively correlated with a) H’ (*R*_*s*_ = − 0.85; *P* < 0.01; Fig. 3A), indicating that this was the golden eagle’s preferred prey in the study area, and b) the consumption (%N) of spur-thighed tortoise (*R*_*s*_ = − 0.75; *P* < 0.01; Fig. 3B). A positive correlation was found between tortoise consumption and H’ (*R*_*s*_ = 0.79; *P* < 0.01; *P* > 0.05 for the rest of the correlations).

### Size, stage and sex selection of the tortoises predated by golden eagles

We identified a minimum of 213 tortoises predated by golden eagles in five territories (T1, T4, T5, T6 and T8). We found a strong linear relationship between FW and weight of living tortoises (*R*^2^ = 0.94), and between FW and shell weight of museum specimens (*R*^2^ = 0.91; Supp. Mat. Fig. S1). The average FW of the 145 preyed individuals that conserved any of the femoral scutes was 63.8 mm (range = 36.4–84.9 mm; average for subadults: 52.7 mm; average for adult males: 59.9 mm; average for adult females: 70.2 mm). According to the abovementioned regressions, the estimated average biomass consumed by eagles was 397.8 g per tortoise (range = 78.1–777.2 g; average for subadults: 238.4 g; average for adult males: 334.6 g; average for adult females 493.7 g; see Table 1 for values at the eagle’s territory level).

**Table 1:** Size (FW = width of femoral scutes) and biomass (total weight, including the shell) of the spur-thighed tortoises predated by golden eagles in SE Spain, according to eagles’ territories with tortoise remains.

Territory	Subadults			Adult males			Adult females			Adults, unknown sex		
	N	FW (mm)	weight (g)	N	FW (mm)	weight (g)	N	FW (mm)	weight (g)	N	FW (mm)	weight (g)
T1	4	51.30 ± 8.33	226.63 ± 95.26	9	55.37 ± 2.89	266.60 ± 36.62	18	69.61 ± 4.02	480.91 ± 70.70	1	74.31	562.61
T4	0	0	0	1	52.84	234.20	0	0	0	0	0	0
T5	23	56.41 ± 6.43	235.13 ± 71.09	14	62.84 ± 9.16	381.51 ± 132.89	46	70.24 ± 5.99	495.05 ± 106.50	2	56.32 ± 4.90	279.58 ± 64.09
T6	5	54.34 ± 3.45	254.02 ± 41.71	5	61.87 ± 4.91	358.04 ± 71.19	11	71.62 ± 7.82	522.30 ± 141.85	1	66.53	427.31
T8	1	56.70	282.79	3	60.94 ± 6.09	345.27 ± 89.53	1	67.51	443.31	0	0	0
All	33	52.73 ± 6.13	328.41 ± 68.32	32	60.10 ± 7.42	337.52 ± 108.04	76	70.26 ± 5.81	494.97 ± 103.99	4	63.37 ± 9.19	398.86 ± 145.64

**Figure 4:**
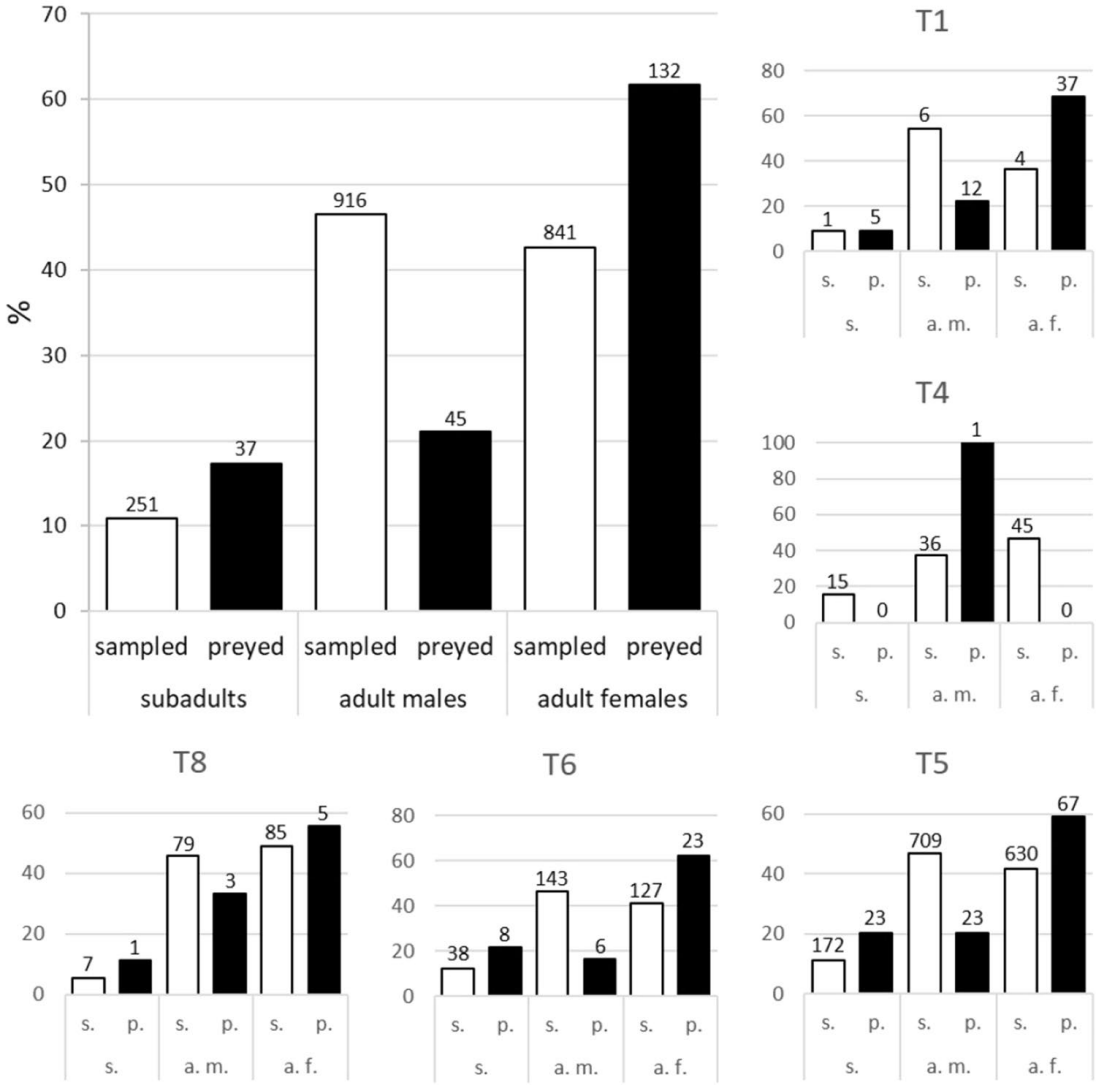
Comparison of the stage and sex structure of the tortoises sampled and predated by golden eagles in the study area. Data are shown for all eagle territories where both sampled and preyed tortoises were available, either pooled (large panel) or separately (small panels). Data for all study years were pooled (see text for more details). The number of tortoises of each class is shown above the bars.

We were able to identify sex and stage in 66.2% of the tortoises predated by golden eagles: 53.9% were adult females, 22.7% adult males and 23.4% subadults (Table 1). Regarding size selection within each tortoise category, eagles preferred the largest subadults (K-W = 17.17; *P* < 0.001) and adult males (K-W = 13.60; *P* < 0.001), but not the largest females (K-W = 1.57; *P* = 0.21; Supp. Mat. Fig. S2). Moreover, we detected a strong selection of adult tortoise females by golden eagles (Fig. 4), both at the eagle population level (*χ*^2^ = 50.92; *P* < 0.01) and at two territories with sufficient sample sizes for preyed and sampled tortoises (*χ*^2^ = 31.10 for T5 and 12.44 for T6; *P* < 0.01 in both cases).

### Predation impact, functional response and effects of rabbit abundance

The estimated size of the total tortoise population was 749,012 individuals (range = 278,749–2,018,642). The abundance of tortoises and rabbits showed wide variation among golden eagle territories (Supp. Mat. Table S2). The golden eagle’s breeding success, used to calculate the kill rates, ranged from 0 to 3 (Table 2). The kill rate ranged from 0 to 73 tortoises per golden eagle breeding season and territory (mean = 18.4, *SD* = 23.4; Table 2). This yielded an average kill rate for the whole study area of 405 tortoises per golden eagle breeding season. Predation rates ranged from 0 to 4.76% (maximum value recorded in T1 within the 2.5 km territorial radius in 2016), with a mean of 0.47% (*SD* = 0.60) for the 2.5 km territorial radius and 0.12% (*SD* = 0.15) for the 5 km territorial radius (Table 2). Predation rates at 2.5 and 5 km territorial radii were highly correlated (*R*_p_^2^ = 0.99, *P* < 0.0001). The overall predation rate for the whole tortoise population and per golden eagle breeding season was 0.12% (range = 0.04–0.32%). The sex ratio of the tortoise population correlated inversely with the predation rate for territories (*R*_s_ = − 0.95, *P* = 0.003; Supp. Mat. Fig. S3), which suggests that golden eagles may be negatively affecting female tortoise abundance.

**Table 2:** Breeding success of golden eagles and kill rates and predation rates by golden eagles on spur-thighed tortoises in SE Spain according to eagle territories, years and territorial radius.

Territory and year (n = 15)	Number of fledglings	Kill rate	2.5 km			5 km		
			Average pred. rate	Min. pred. rate	Max. pred. rate	Average pred. rate	Min. pred. rate	Max. pred. rate
T1 2014	2	42	1.45	0.53	3.92	0.38	0.14	1.02
T1 2016	1	51	1.76	0.65	4.76	0.46	0.17	1.24
T1 2017	1	0	0	0	0	0	0	0
T2 2014	2	0	0	0	0	0	0	0
T3 2016	3	0	0	0	0	0	0	0
T4 2014	0	3	0.03	0.01	0.08	0.01	< 0.01	0.03
T4 2016	2	9	0.09	0.03	0.26	0.03	0.01	0.09
T5 2015	2	41	1.17	0.44	3.11	0.31	0.11	0.82
T5 2017	1	28	0.80	0.30	2.12	0.21	0.07	0.56
T6 2014	1	73	0.82	0.31	2.18	0.22	0.08	0.59
T7 2015	1	9	0.09	0.03	0.26	0.03	0.01	0.09
T8 2014	1	20	0.33	0.12	0.87	0.11	0.04	0.29
T9 2014	1	0	0	0	0	0	0	0
T10 2014	2	0	0	0	0	0	0	0
T11 2014	2	0	0	0	0	0	0	0

**Figure 5:**
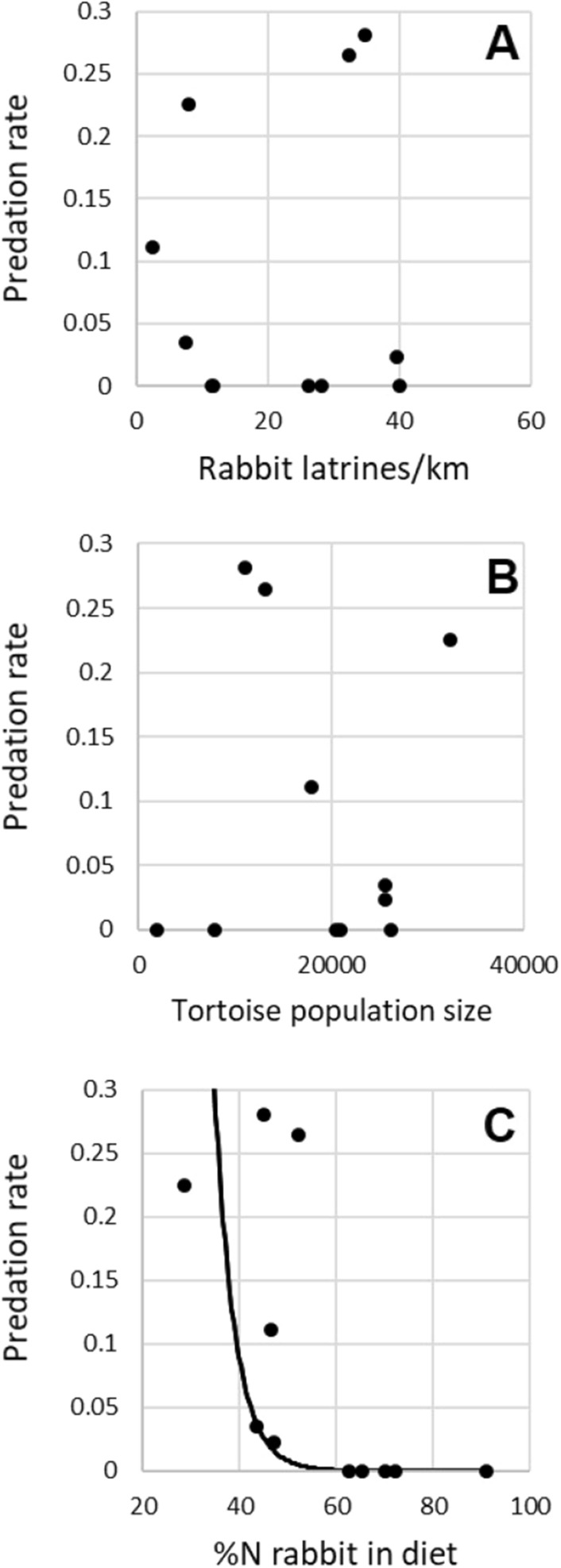
Relationships between predation rate on spur-thighed tortoise by golden eagle and (**A**) tortoise abundance within the 5 km eagle territorial radius (functional response), (**B**) %N of rabbit in eagles’ diet, and (**C**) rabbit abundance index. n = 11 golden eagle territories with sufficient diet data (for territories with > 1 year sampled, mean values are represented). Only case (**B**)showed a significant relationship (*R*^2^ = 0.72, *P* = 0.001). Territories T1 and T5 (see text) are shown in case (**C**).

We did not find any functional response of golden eagles to tortoise (Fig. 5A) or rabbit abundance (Fig. 5B), but we observed a negative exponential relationship (*R*^2^ = 0.72, *P* = 0.001) between the predation rate of tortoises and the consumption of rabbits. In particular, the predation rate of tortoises was always 0 when eagles included more than 60%N of rabbits in their diet (Fig. 5C). Although we did not find a clear relationship between rabbit abundance and predation rate of tortoises, an exponential negative relationship arises if two territories (T1 and T5) are excluded (*R*^2^ = 0.72, *P* = 0.001).

## Discussion

The spur-thighed tortoise played a secondary role in the golden eagle diet in our study area. Approximately half of the studied golden eagle breeding pairs killed tortoises (6 out of 11), although there was wide variation among eagle territories, with one territory (T6) in which tortoises were the most frequently taken prey (Fig. 2). In other Palearctic areas where golden eagles and *Testudo* tortoises coexist, tortoise consumption by this raptor is more frequent (53–93%N)^24–29,48^. The presence in Mediterranean Spain of a key medium-sized prey species, namely the Eurasian rabbit^49^, may be behind the relatively low representation of tortoises in the golden eagle’s diet in our study area. Despite myxomatosis and RHD outbreaks, rabbits are still the golden eagle’s staple prey in our study area. As revealed by the observed negative relationship between eagles’ trophic diversity and rabbit consumption (see^50,51^, golden eagles generally preferred rabbits over other prey, which has been previously indicated in other Iberian areas ^21,44^ and for other Iberian predators^45–47,52–54^. The average rabbit densities, recalculated from our latrine surveys (see^55^), were low (< 1 ind./ha) to moderate (3–4 ind./ha) in most territories, though we detected some high-density patches (> 7 ind./ha). Our results suggest that these rabbit densities are, in general, sufficient for golden eagles to prefer rabbits over other prey, including tortoises.

The observed negative relationship between the consumption (%N in diet and predation rate) of tortoises and the consumption (%N) of rabbits suggested that spur-thighed tortoises are an alternative prey to rabbits for golden eagles (Fig. 3B). However, we found that the predation rates on tortoises did not always match the levels expected based on rabbit abundance (i.e. we found two golden eagle pairs that captured more tortoises than expected), which suggests that factors other than rabbit abundance could affect the eagle-tortoise relationship. This idea is also supported by the fact that the average density of tortoises and rabbits within golden eagle territories was similar (2.3 and 2.2 ind./ha, respectively). For instance, habitat structure can affect prey accessibility for raptors^56^. Also, individual variation in prey preferences and hunting ability may play a role^57^, especially considering the elaborate hunting technique that is required to feed on tortoises^19^. Even though tortoises are easier to capture than rabbits, the latter might be a more profitable prey because manipulation costs are much higher for tortoises than for rabbits, as eagles must transport each captured tortoise to an optimal site, where they break the shell by dropping the tortoise on a rocky slope several times^20,26^. Moreover, the average biomass gained is much higher for rabbits (786 g) than for tortoises (387 g). Thus, according to the Optimal Diet Theory framework^58^, rabbits might be a more optimal prey than tortoises, which would be selected only when their abundance is much higher than that of rabbits. In this context, the lack of golden eagles’ functional response to tortoises was an expected outcome. Also, our findings encourage further exploration of the effects of optimal and alternative prey availability in golden eagle productivity^59^.

Although our results may suggest a low limiting potential of golden eagles on the studied tortoise population, we estimated the minimum number of tortoises eaten, so actual kill rates could be higher. Moreover, golden eagles selected the largest tortoises, particularly adult females, which could affect tortoise population dynamics. These prey size and sex preferences also agree with the Optimal Diet Theory^58^ for two reasons. On the one hand, selecting large tortoises maximises the benefits obtained in terms of edible biomass in relation to the costs of tortoise manipulation. On the other hand, adult spur-thighed tortoise females seem to select places with less shrub cover than males^60^, thus making adult females particularly vulnerable to predation from aerial predators such as golden eagles. The population dynamics of tortoises are supported by high (and relatively stable) values of adult survival rates^16,17^. Indeed, even small reductions in this demographic parameter (e.g. 1–2%) can reduce the population growth rate of spur-thighed tortoises and drive the local populations to extinction^17,36,61^. In addition, we found that the tortoise population sex ratio was influenced by predation by golden eagles, with sex ratio being biased towards males in those eagle territories with higher predation rates (Fig. 4). This contrasts with the sex ratio of the whole tortoise population studied, which is quite constant (1:1)^17^. Biased sex ratios may reduce mate-finding efficiency, to which the population dynamics of the spur-thighed tortoise is very sensitive^17^. Therefore, the negative effects on tortoise demography of the predation exerted by golden eagles are expected to be higher compared to a scenario where eagles randomly capture tortoises, irrespective of their size, stage and sex.

The predation pressure by golden eagles on spur-thighed tortoises found in this study is likely the highest impact ever found, given the historical population trends of rabbits and golden eagles, and past contributions of the former to the diet of the latter. Though there is no dietary information for golden eagles before the myxomatosis outbreak, data from the end of the twentieth century obtained in different areas of Mediterranean Spain (including SE Spain) indicated that rabbits usually represented more than 50%N of the golden eagle’s diet^62^. As can be seen in Fig. 3B, the predation impact on tortoises is extremely low above this level of rabbit consumption. The myxomatosis and RHD outbreaks in the mid- and late-twentieth century, respectively, led to a strong reduction in rabbit abundance throughout its distribution range in Mediterranean Spain, and current rabbit abundance is still far from pre-outbreak levels^43^. In the second half of the twentieth century, golden eagles in Spain were intensively persecuted through poisoning and shooting, a practice that was even rewarded by the government^63^. However, in the present century, legal protection and associated conservation measures have led to a great recovery of the golden eagle Iberian population^62,64^. In the current scenario of decreased abundance of the primary prey (the European rabbit) and increased abundance of the predator (the golden eagle), our findings reveal the key role of rabbits in governing the predation pressure of golden eagles on tortoises. Further research should investigate whether the enhanced predation on tortoises in the patches (i.e. golden eagle territories) with lower abundance and consumption of rabbits affects the tortoise population dynamics. Hyperpredation by rabbit predators (including golden eagles) due to rabbit outbreaks has already been shown to drive population declines of other alternative prey to rabbits, such as red-legged partridges, on a large scale in Spain^13,14^.

Spur-thighed tortoises are threatened by climate change, habitat loss and fragmentation, pet collection and trade, wildfires and emerging diseases^40,41,61,65^. Hyperpredation by golden eagles is an additional cause of mortality that could hamper the long-term persistence of the tortoise population, at least at the intra-population scale (i.e. within some golden eagle territories), where predation impact may be up to 4%. Future monitoring and studies should determine to what extent predation by golden eagles is additive or compensatory to other mortality causes, and whether the loss of tortoises through predation may be compensated for by immigration from outside the golden eagle territories. Also, the role of learning in the ability of individual golden eagles to handle tortoises deserves further investigation.

Our results deserve attention from wildlife managers, especially taking into account the rabbits’ slow recovery after outbreaks and increases in the golden eagle Iberian population^62,64^. Several applied recommendations may arise from our study. First, studies based on population viability analyses^66^, which should include hyperpredation effects of golden eagles at different spatial scales and ecological scenarios (e.g. new rabbit outbreaks), are necessary to reveal the effect of golden eagle predation on the long-term demography of this relict spur-thighed tortoise population. Second, detailed monitoring of tortoises, rabbits and eagles should be undertaken within the tortoise population range. Third, rabbits are the main small game species in Spain^43^, including in our study area. Thus, hunting of rabbits should be managed within the spur-thighed tortoise range in SE Spain, especially in areas of low rabbit density. Fourth, vigilance should be encouraged in golden eagle territories with low rabbit abundance to minimise other threats to tortoises, such as wildfires and habitat degradation. In addition, our results may inspire research in other areas where golden eagles frequently capture tortoises, as well as in other systems with similar predator and/or primary prey dynamics. Overall, we have shown how population changes of predators and key prey species can impact the conservation of alternative prey species through emerging inter-specific interactions, which are a common outcome of global environmental change and an increasing challenge for conservationists^67^.

## Material and methods

### Study area and golden eagle population

The study area comprises the whole range of the spur-thighed tortoise in SE Spain (3,195 km^2^ in Murcia and Almeria provinces; Fig. 2). The climate is semiarid Mediterranean with annual rainfall ranging from 200 to 570 mm. The relief is formed by chains of small mountains (up to 1,247 m a.s.l.) and plains. Natural vegetation is usually limited to these mountain ranges, with scrublands of *Stipa tenacissima* and *Anthyllis cytisoides* and pine forests (*Pinus halepensis*), both native and planted. Plains are occupied by irrigated and non-irrigated crops. The current local golden eagle population consists of 22 breeding pairs (Fig. 2), most of which nest on cliffs. This means an increment of 16 pairs from 2002 (authors’ unpublished data). According to official hunting bags of the Murcia Region, current rabbit densities in the study area are 25–30% the pre-RHD outbreak levels^43^.

### Golden eagle diet and prey preferences

The golden eagle’s diet was studied in 11 territories during four breeding seasons (2014–2017) by analysing regurgitated pellets, which were collected at perching sites on or close to breeding cliffs at the end of the breeding season, usually June and July (e.g.^23,44^). Eight territories were visited in 2014 (T1, T2, T4, T6, T8, T9, T10 and T11), two in 2015 (T5 and T7), three in 2016 (T1, T3 and T4) and two in 2017 (T1 and T5). Following Real^68^, each prey species identified in one food pellet was counted as one individual. Data were considered representative of eagles’ diet when at least 20 preys were obtained for a given pair and year^47^. The diet data are shown in terms of relative frequency (%N) and relative ingested biomass (%B), which were calculated by assigning the corresponding species average weight to each prey individual^68^, with some exceptions. Rabbits’ bone sizes served to estimate the biomass contribution of rabbits and classify them as: juvenile (250.0 g), subadults (637.5 g) and adults (1062.5 g)^69^. For tortoises, we estimated average weight (shell excluded) according to the size of shells collected in eagles’ roosts located on or near breeding cliffs (see below). A biomass contribution of 2 kg (i.e. roughly the maximum intake of a golden eagle pair with two chicks for two days^19^) was assigned to prey species weighing > 4 kg, namely juvenile wild boars and mammalian carnivores (all of these preys were anecdotal in the eagle’s diet; see the Results). For snakes and lizards, we took the maximum weight of each species, as all the preyed individuals were large (see Supp. Mat. AppendixS1 for details of prey biomass calculations). Different prey species were categorised into the following six prey groups: rabbit, other mammals, red-legged partridge, other birds, tortoises, other reptiles.

To identify alternative prey species (i.e. those whose consumption is inversely related), we carried out pair-wise Spearman’s rank correlations for all prey categories (%N), with territory as the sample unit. We then calculated the Shannon–Weaver’s index of trophic diversity (H’) based on the relative frequency of these prey groups in the eagle’s diet (%N), for each eagle territory with sufficient diet data (i.e. > 20 prey). To assess prey preferences (i.e. prey whose representation in the predator’s diet is inversely related to trophic diversity were considered preferred prey^50,51^), we conducted Spearman’s rank correlations between the relative frequency of each prey category in the eagle’s diet (%N) and H’, for each eagle territory with sufficient diet data.

### Tortoise densities and population structure

The spur-thighed tortoise’s life cycle includes three stages: juveniles (with soft shells, 0–4 years old), subadults (with ossified shells, 5–8 years old), and adults (> 8 years old, sexable individuals). We estimated the population size 1) of the whole tortoise population in SE Spain and 2) within each eagle territory using a previously developed abundance model for the species^70^. A negative binomial GLM model explaining the abundance index and a linear regression between the abundance index with the known values of the species density (obtained by means of field transects^71^) provided a spatial model of abundance at 1 × 1 km resolution. This model allows absolute estimates of density and confidence intervals in each 1 × 1 km cell. The tortoise density estimates in each 1 × 1 km cell were used to compute the absolute abundance (total number of individuals, including juveniles, subadults and adults) for the whole study area and for each golden eagle nest. For this purpose, we used two theoretical circular territories around the territory centre (i.e. the arithmetic centre of all the nests within a given territory that were occupied during the study period): a) radius of 5 km (which approximately represents the average minimum distance between neighbouring pairs of golden eagles in the study area) and b) radius of 2.5 km (half the previous radius; see Moleón^69^ for a similar approach). All 1 × 1 km cells with > 50% of the surface within the study area or within each territory buffer, respectively, were included (for more details, see Supp. Mat. Appendix S2 and Table S1, and Esteve-Selma^70^).

The tortoise population structure data were obtained from the Department of Applied Biology database (Univ. Miguel Hernández, Spain). This includes 36 1 × 1 km tortoise sampling locations within golden eagle territories. At these sites, 2760 individuals were sampled during the monitoring activities performed in spring 2005–2016. Information in this database includes biometrical measures, such as femoral width (FW; width of femoral scutes) and weight, stage and sex. Adults were sexed according to secondary characters and size^36^.

### Size, stage and sex selection of the tortoises predated by golden eagles

We collected remains of tortoise shell in eagles’ roosts located on or near breeding cliffs to estimate the number, stage, sex and biomass of the tortoises predated by eagles. First, we estimated the minimum number of individuals captured per territory and year by quantifying the number of repeated shell elements. Second, we used FW as a proxy for the size of these individuals^72^. FW was the measure most frequently obtained from shell remains (44.5% of shells). From a sample of 2008 living tortoises in the study area with biometrical information for the period 2005–2016, we built a linear regression between FW and weight. Then, from a sample of 104 complete shells from the zoological collection held in the Dept. of Applied Biology (Univ. Miguel Hernández, Spain), we built a linear regression between FW and shell weight. Therefore, we were able to estimate the biomass of each predated tortoise individual after excluding the shell. Third, stage and sex were assigned to each individual according to FW and physical divergences, respectively ^72^.

Size (i.e. FW) selection was studied by pooling the entire sample (all years and eagle territories), as the number of measured tortoise individuals was insufficient to make temporal and spatial distinctions. We used a Kruskal–Wallis test to compare the size of the tortoises available in the field and those predated by eagles, separately for each category (subadults, adult males and adult females). To study the selection of tortoise stage and sex by golden eagles, we used Chi-square tests to compare the distribution of the three classes in the field vs. in the pool of predated tortoises. Chi-square tests were conducted for all the golden eagle territories together, and separately for each of the two territories with sufficient data for predated tortoises and tortoises sampled from the database (i.e., more than 10 individuals per group) within a 5 km radius from territory centres (all years pooled in both cases).

### Kill and predation rates, functional response and effects of rabbit abundance

We estimated the predation impact of the golden eagle’s breeding units (adults plus nestlings) on the tortoise populations in the study area throughout the eagles’ breeding season (March to July, c. 124 days, including 44 days for incubation and 80 days for the nestling period in the study area; authors’ unpublished data), using an adaptation of the formula by Linden and Wikman^69,73^:

$${\text{NP }} = \, \left[ {\left( {{\text{CF }} + {\text{ CM }} + {\text{ CY}}} \right){\text{ PPB}}} \right] \, /{\text{ PW}}$$

where NP is the number of tortoises captured by eagles, CF is females’ consumption (number of females x study period length x female daily dietary requirements), CM is males’ consumption (number of males x study period length x male daily dietary requirements), CY is nestlings’ consumption (number of nestlings x nestling consumption), PPB is the proportion of prey (tortoise) biomass in the eagle’s diet, and PW is the corrected average prey weight. PW represents the mean usable biomass of tortoises (i.e. 387 g; see the Results). Food intakes of 96 kg/year and 112 kg/year were respectively assumed for the dietary needs of an adult male and female golden eagle^74^, which respectively represents 8.0 and 9.3 kg/month. Hence, the total intake during the golden eagle breeding period in SE Spain was 32.0 kg for males and 37.2 kg for females. For nestlings, we used the data provided by Collopy^75^, who estimated an average food consumption for wild female and male nestlings of 309.8 and 278.5 g/day, respectively. For each territory and year, we calculated the breeding success as the number of > 60 day-old fledglings (n = 15 breeding attempts accurately monitored). The golden eagle’s breeding season overlaps with the main activity period of tortoises in SE Spain^60^, from March to June. In summer (July–August) and winter (November–February) tortoises are inactive and retire into refuges, while their activity during fall (September–October) is very limited^17,60^. Thus, our estimations would be close to the actual annual predation impact.

The impact results are presented as the number of tortoises consumed by eagles, or ‘kill rate’ (i.e. NP in the formula above), and the percentage of the tortoise population consumed by eagles, or ‘predation rate’^69^. Both the kill and predation rates were estimated on two spatial scales: for the entire study area and for each golden eagle territory (11 territories with sufficient diet data; Fig. 2). In the latter case, predation rate was calculated according to the tortoise abundance within the 2.5 and 5 km radii around golden eagle nests. Three predation rate estimates were calculated: 1) mean predation rate, obtained from the most probable tortoise population size in the abundance model, 2) minimum predation rate, from the estimated maximum tortoise population size, and 3) maximum predation rate, from the estimated minimum tortoise population size. In addition, to determine if golden eagles affect the tortoise sex ratio in the study area, we explored the relationship between the population sex ratio of tortoises (females:males) and predation rate at the eagle territorial level (5 km territory radius), using Spearman’s rank correlations. To calculate the tortoise sex ratio, we use the available data about tortoise population structure in the study area, we obtained a sufficient sample size (more than 10 individuals) for six territories (T3, T4, T5, T6, T7 and T8).

We explored the functional response of eagles to tortoise density by relating the predation rate to tortoise abundance in the territories with sufficient diet data (n = 11; 5 km territorial radius;^51^). Moreover, we studied the effects of rabbit abundance and consumption by golden eagles on the predation rates of tortoises at the eagle territory level (5 km radius). Rabbit abundance was estimated through latrine counting, a good indicator of rabbit density^55^, for the 11 golden eagle territories with sufficient diet data. We carried out four 1 km walking transects distributed within the 2.5 km radius around each occupied nest, in July and August 2019, i.e. immediately after the rabbit population reached its seasonal peak in the study area^76^. Also, this avoided the main rainfall season, thus minimising biases associated with latrine losses. Importantly, rabbit populations in our study area do not undergo cyclic fluctuations, and no major RHD outbreak occurred in our study area during the study period. Transects were carried out following unpaved roads and trails on scrublands, which are the main habitat for this rabbit population. We always kept away from ecotones to avoid overestimations. Rabbit abundance was expressed as latrines/km. Using the eagle territories as the sample unit, we used Spearman’s rank correlations to explore the relationship between the predation rate of tortoises and (1) the consumption (%N) of rabbits and (2) the mean abundance index of rabbits. Although latrine sampling was delayed compared to diet sampling, based on the official hunting bags of the Murcia Region, there was spatial homogeneity in interannual rabbit abundance variations, which allowed comparisons among golden eagle territories.

### Ethics approval

All methods and procedures were performed in accordance with the relevant guidelines and regulations, followed the protocols approved by Universidad Miguel Hernández (UMH) Ethics Committee (OIR) and were authorized by the Dirección General de Gestión del Medio Natural of Andalusian Government (SGB/FOA/AFR) and the Dirección General de Medio Natural of the Murcia Region for golden eagle territories (AUF20140061) and the spur-thighed tortoise populations (SGYB/AF/DBP, SGYB/AFR/DBP, AUF20160056, AUF20140057). All methods are reported in accordance with ARRIVE guidelines.

## Supplementary Information

Supplementary Information.

## Data Availability

All data generated or analysed during this study are included in this published article and its supplementary information files.
