# Supplementary material for: Predation impact on threatened spur-thighed tortoises by golden eagles when main prey is scarce
Source: Sci Rep. 2022 Oct 25;12:17843. doi: 10.1038/s41598-022-22288-9 (PMC9596414; doi:10.1038/s41598-022-22288-9)

Supplementary files for

**Predation impact on threatened spur-thighed tortoises by golden eagles when main prey is scarce**

José M. Gil-Sánchez, Roberto C. Rodríguez-Caro, Marcos Moleón, María C. Martínez-Pastor, Mario León-Ortega, Sergio Eguia, Eva Graciá, Francisco Botella, José A. Sánchez-Zapata, Julia Martínez-Fernández, Miguel A. Esteve-Selma, Andrés Giménez

This material includes:

**Appendix S1.** Estimation of the biomass consumed by golden eagles**.**

**Appendix S2.** Construction method of the of spur-thighed tortoise abundance model**.**

**Table S1.** Golden eagle diet in the study area.

**Table S2.** Absolute abundance values of spur-thighed tortoise in the 2.5 and 5 km buffer areas around the golden eagle nests in the study area.

**Figure S1.** Regressions for the size estimations of predated tortoises.

**Figure S2.** Comparison of the average size of spur-thighed tortoises available and preyed in the 11 golden eagle territories, separately for each tortoise stage and sex class.

**Figure S3.** Relation between the sex ratio of tortoises and predation rate in six golden eagle territories.

**Appendix S1. Estimation of the biomass consumed by golden eagles, according to prey species/group.**

**European rabbit:**

Three stages were considered for rabbits: juveniles (mean weight: 250 g), subadults (750 g) and adults (1,250 g; Donázar, 1989; Gil-Sánchez et al., 1999). Juvenile individuals were normally eaten completely by eagles, while 15% of the average weight was deducted in relation to the biomass not eaten by subadults and adults; thus, the final weights used for these two stages were 637.5 and 1,062.5 g, respectively. We aged 88 rabbits eaten by eagles: 11.4% juveniles, 43.2% subadults and 45.4% adults. Consequently, the mean weight of the rabbits consumed by golden eagles in our study area during the breeding season was 786.6 g.

**Red-legged partridge:**

According to Moleón et al. (2011), the corrected mean weight (i.e. without the parts normally rejected by eagles) of red-legged partridges was 359.6 g.

**Other mammals:**

For Eurasian badger (*Meles meles*) and wild boar (*Sus scrofa*), we assigned a weight of 2,000 g (only juvenile individuals of these species were preyed by golden eagles in our study). For rats (*Rattus* spp.), we took a weight of 250 g (Aulegnier et al., 2009).

**Other birds:**

According to Moleón et al. (2011), the corrected mean weight of pigeons was 338.7 g. For hens, we assigned a weight of 2,000 g. In the case of individuals of other bird species, we took an average weight of 405.1 g (Moleón et al., 2011).

**Other reptiles:**

For snakes (*Elaphe scalaris*, *Malpolon monpessulanun*), we used a weight of 1,000 g. Ocelated lizards (*Timon ocellatus*) were considered to weight 500 g (Gil and Pleguezuelos, 2011).

**References**

Aulegnier, S., Haffner, P., Mitchell-Jones, A. J., Moutou, F. & Zima, J. (2009). *Guía de los mamíferos de Europa, del norte de África y de Oriente Medio*. Lynx Editions, Barcelona.

Donázar, J. A. (1989). Variaciones geográficas y estacionales en la alimentación del búho real (*Bubo bubo*) en Navarra. *Ardeola* 36: 25-39.

Gil, J.M. & Pleguezuelos, J.M. 2001. Prey and prey-size selection by the short-toed eagle (*Circaetus gallicus*) during the breeding season in Granada (south-eastern Spain). *J. Zool*. 255: 131-137

Gil-Sánchez, J. M., Valenzuela, G. & Sánchez, J. F. (1999). Iberian wild cat *Felis silvestris tartessia* predation on rabbit *Oryctolagus cuniculus*: functional response and age selection. *Acta Theriol.* 44: 421-428.

Moleón, M., Sánchez-Zapata, J. A., Gil-Sánchez, J. M., Barea-Azcón, J. M., Ballesteros-Duperón, E. & Virgós, E. (2011). Laying the foundations for a human-predator conflict solution: Assessing the impact of Bonelli’s eagle on rabbits and partridges. *PLoS ONE* 6: e22851.

**Appendix S2. Construction method of the spur-thighed tortoise abundance model.**

The spur-thighed tortoise (*Testudo graeca*) population size for the entire study area and for each golden eagle territory was estimated from a spatial habitat-abundance model (Esteve-Selma et al. in press). Local Ecological Knowledge (LEK), specifically interviews with shepherds, was applied to obtain a tortoise abundance index (see Anadon et al., 2009 for details) in 1.215 1x1 km cells. The environmental database at the 1 km^2^ resolution provided climate information (22 annual, seasonal and extreme value variables), as well as local factors, specifically topographic (slope and aspect on two spatial scales), lithologic (six classes on two spatial scales) and land-use variables (eight land cover classes on two spatial scales).

The favorable climate area of the species was defined by developing a distribution model using presence-absence data (65% explained deviance). The ROC (Receiver Operating Characteristic) curve identified the value of 20% presence probability as the threshold by defining the favorable climate area (5,279 1x1 km cells). In this area, a negative binomial GLM model explaining the abundance index was built. The resulting model (11 explanatory variables, 32.74% explained deviance) shows the rejection by tortoises of irrigated-tree crops and a quadratic response to the number of frost days, winter temperature, autumn temperature and drylands on a 3x3 km scale.

The absolute density estimates and confidence intervals in each 1x1 km cell were obtained by applying linear regression between the abundance index and the known values of the species density obtained through field transects (Anadón et al., 2009). This gave three absolute abundance values: the fitted, minimum and maximum values of the prediction interval of regression.

The tortoise density estimates in each 1x1 km cell were used to compute the absolute abundance (total number of individuals) for the whole distribution range and in a circular buffer around each golden eagle nest on two scales: 2.5 and 5 km radii. Only those 1x1 km cells with more than 50% of the surface inside the calculation area were included. The total population size for the complete distribution range of tortoises was 749,012 individuals (range of estimation = 278,749–2,018,642). The absolute abundance estimates in the real eagle territories ranged from 0 to 10380 tortoises for the 2.5 km radius buffers, and between 1867 and 26245 for the 5 km radius buffers (Table 1).

**References**

Anadón, J. D., Giménez, A., Ballestar, R. and Pérez, I. (2009). Evaluation of local ecological knowledge as a method for collecting extensive data on animal abundance. *Conserv. Biol*. 23: 617-625.

Esteve-Selma, M. A., Martínez-Fernández, J., Montoya-Bernabeu, P., Rodríguez-Caro R.C., Graciá E & Giménez A. (in press). Effects of climate change on the potential distribution of Testudo graeca in southeastern Iberian Peninsula. In Graciá E., Rodríguez-Caro R.C. and Giménez A. Conservation of Mediterranean tortoises under global change. Madrid. Asociación Herpetológica Española. ISBN: 978-84-921999-6-9

**Table S1. Golden eagle diet data in the study area, expressed as the number of prey individuals (N), frequency of prey individuals (%N) and relative ingested biomass (%B).**

| **Territory and year** | **N pellets** | **Rabbit** | **Other mammals** | **Partridge** | **Other birds** | **Tortoise** | **Other reptiles** | **Total** |
| --- | --- | --- | --- | --- | --- | --- | --- | --- |
| **N** | | | | | | | | |
| T1 2014 | 43 | 36 | 5 | 13 | 11 | 21 | 4 | 90 |
| T1 2016 | 16 | 12 | 0 | 4 | 1 | 9 | 2 | 28 |
| T1 2017 | 13 | 11 | 0 | 4 | 5 | 0 | 1 | 21 |
| T2 2014 | 44 | 43 | 0 | 5 | 12 | 0 | 6 | 66 |
| T3 2016 | 36 | 33 | 3 | 6 | 1 | 0 | 4 | 47 |
| T4 2014 | 16 | 14 | 25 | 5 | 0 | 2 | 2 | 48 |
| T4 2016 | 47 | 44 | 2 | 10 | 2 | 4 | 6 | 68 |
| T5 2015 | 51 | 42 | 3 | 5 | 7 | 21 | 10 | 88 |
| T5 2017 | 38 | 33 | 0 | 6 | 8 | 11 | 0 | 58 |
| T6 2014 | 20 | 10 | 1 | 6 | 2 | 15 | 1 | 35 |
| T7 2015 | 44 | 33 | 8 | 10 | 14 | 5 | 6 | 76 |
| T8 2014 | 39 | 33 | 0 | 8 | 4 | 10 | 16 | 71 |
| T9 2014 | 25 | 20 | 2 | 1 | 7 | 0 | 2 | 32 |
| T10 2014 | 20 | 20 | 0 | 1 | 1 | 0 | 0 | 22 |
| T11 2014 | 44 | 44 | 2 | 7 | 4 | 0 | 4 | 61 |
| **%N** | | | | | | | | |
| T1 2014 |  | 40.01 | 5.56 | 14.44 | 12.22 | 23.33 | 4.44 |  |
| T1 2016 |  | 42.85 | 0 | 14.28 | 3.57 | 32.14 | 7.14 |  |
| T1 2017 |  | 52.38 | 0 | 19.04 | 23.81 | 0 | 4.76 |  |
| T2 2014 |  | 65.15 | 0 | 7.58 | 18.18 | 0 | 9.09 |  |
| T3 2016 |  | 70.21 | 6.38 | 12.76 | 2.12 | 0 | 8.51 |  |
| T4 2014 |  | 29.17 | 52.07 | 10.42 | 0 | 4.17 | 4.17 |  |
| T4 2016 |  | 64.70 | 2.94 | 14.70 | 2.94 | 5.88 | 8.82 |  |
| T5 2015 |  | 47.72 | 3.40 | 5.68 | 7.95 | 23.86 | 11.36 |  |
| T5 2017 |  | 56.89 | 0 | 10.34 | 13.79 | 18.96 | 0 |  |
| T6 2014 |  | 28.57 | 2.86 | 17.14 | 5.71 | 42.86 | 2.86 |  |
| T7 2015 |  | 43.42 | 10.52 | 13.15 | 18.42 | 6.57 | 7.89 |  |
| T8 2014 |  | 46.48 | 0 | 11.27 | 5.63 | 14.08 | 22.54 |  |
| T9 2014 |  | 62.49 | 6.25 | 3.13 | 21.88 | 0 | 6.25 |  |
| T10 2014 |  | 90.90 | 0 | 4.55 | 4.55 | 0. | 0 |  |
| T11 2014 |  | 72.12 | 3.28 | 11.48 | 6.56 | 0. | 6.56 |  |
| **%B** | | | | | | | | |
|  |  |  |  |  |  |  |  |  |
| T1 2014 |  | 50.26 | 13.31 | 8.29 | 7.90 | 14.82 | 5.38 |  |
| T1 2016 |  | 57.62 | 0 | 8.78 | 2.47 | 21.85 | 9.26 |  |
| T1 2017 |  | 67.20 | 0 | 11.17 | 15.73 | 0 | 5.89 |  |
| T2 2014 |  | 75.10 | 0 | 3.99 | 10.79 | 0 | 10.10 |  |
| T3 2016 |  | 71.99 | 12.49 | 5.98 | 1.12 | 0 | 8.41 |  |
| T4 2014 |  | 20.92 | 71.26 | 3.41 | 0 | 1.51 | 2.88 |  |
| T4 2016 |  | 71.86 | 6.22 | 7.46 | 1.68 | 3.30 | 9.45 |  |
| T5 2015 |  | 56.85 | 7.74 | 3.09 | 4.87 | 14.37 | 13.05 |  |
| T5 2017 |  | 72.64 | 0 | 6.03 | 9.07 | 12.24 | 0 |  |
| T6 2014 |  | 41.27 | 7.87 | 11.32 | 4.25 | 31.30 | 3.98 |  |
| T7 2015 |  | 48.27 | 22.31 | 6.68 | 10.54 | 3.69 | 8.46 |  |
| T8 2014 |  | 55.73 | 0 | 6.17 | 3.47 | 8.54 | 26.06 |  |
| T9 2014 |  | 67.10 | 12.79 | 1.53 | 12.09 | 0 | 6.47 |  |
| T10 2014 |  | 95.36 | 0 | 2.17 | 2.45 | 0 | 0 |  |
| T11 2014 |  | 77.28 | 6.69 | 5.62 | 3.61 | 0 | 6.77 |  |

**Table S2. Absolute abundance values (number of individuals) of spur-thighed tortoise (*Testudo graeca*) in the 2.5 and 5 km buffer areas around the golden eagle nests in the study area.** Fitted, maximum and minimum estimates are presented. See Appendix S2 and main text for details.

| **Golden eagle territory** | **Absolute abundance of *T. graeca* in a 2.5 km buffer area around nests** | | | **Absolute abundance of *T. graeca* in a 5 km buffer area around nests** | | |
| --- | --- | --- | --- | --- | --- | --- |
|  | **Fitted value** | **Maximum value** | **Minimum value** | **Fitted value** | **Maximum value** | **Minimum value** |
| T1 | 2886 | 7791 | 1071 | 11002 | 29616 | 4094 |
| T2 | 2263 | 5957 | 860 | 7882 | 21125 | 2947 |
| T3 | 10380 | 27666 | 3896 | 26245 | 69689 | 9890 |
| T4 | 9047 | 23845 | 3433 | 25564 | 67636 | 9665 |
| T5 | 3481 | 9211 | 1316 | 13211 | 35122 | 4974 |
| T6 | 8834 | 23374 | 3339 | 32332 | 85673 | 12204 |
| T7 | 9139 | 24161 | 3457 | 25531 | 67355 | 9679 |
| T8 | 5982 | 15729 | 2275 | 17896 | 47386 | 6764 |
| T9 | 12146 | 32683 | 4516 | 20880 | 56068 | 7785 |
| T10 | 5779 | 15217 | 2195 | 20433 | 54672 | 7643 |
| T11 | Beyond the climatic range of *T. graeca* | | | 1867 | 4949 | 705 |

**Figure S1. Regressions for the size estimations of predated tortoises.** Relations between the femoral scutes width (FW) and weight of living tortoises (2131 living tortoises) and tortoise shells (104 shells from the zoological collection; see main text for details).


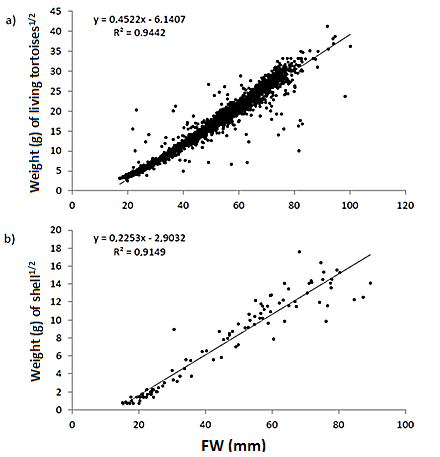


**Figure S2. Comparison of the average size (femoral scutes width, FW) of spur-thighed tortoises available and preyed in the 11 golden eagle territories of the study area with sufficient diet sample size, separately for each tortoise stage and sex class.** Data for all study years and territories were pooled (see main text for more details).

**
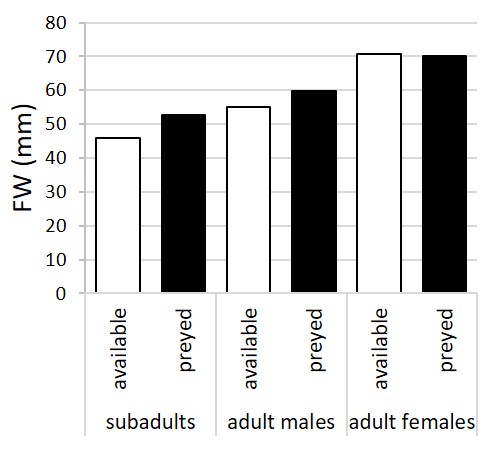
**

**Figure S3 Relation between the sex ratio of tortoises and their predation rate in six golden eagle territories (T3, T4, T5, T6, T7, T8).**


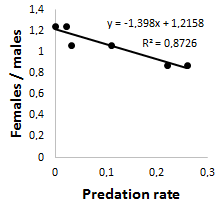

Supplement: Supplementary file 1 — Supplementary Information. [file 41598_2022_22288_MOESM1_ESM.docx]
